# Eco-physiological basis of shade adaptation of *Camellia nitidissima*, a rare and endangered forest understory plant of Southeast Asia

**DOI:** 10.1186/s12898-018-0159-y

**Published:** 2018-02-07

**Authors:** Shengfeng Chai, Jianmin Tang, Azim Mallik, Yancai Shi, Rong Zou, Jitao Li, Xiao Wei

**Affiliations:** 1 Guangxi Key Laboratory of Plant Conservation and Restoration Ecology in Karst Terrain, Guangxi Institute of Botany, Guangxi Zhuang Autonomous Region and Chinese Academy of Sciences, Guilin, 541006 China; 2 Department of Biology, Lakehead University, Thunder Bay, ON P7B 5E1 Canada

**Keywords:** *Camellia nitidissima*, Light gradient, Photosynthetic light response, Chlorophyll fluorescence, Physiology, Photoinhibition

## Abstract

**Background:**

*Camellia nitidissima*, a rare and endangered shrub is narrowly distributed in South China and North Vietnam occurring in forest understory. Their light tolerance mechanism is unclear. We measured photosynthesis and related parameters on 2-years-old cuttings growing at 10, 30, 50 and 100% sunlight. Our research question was: At what light level are *C*. *nitidissima* cuttings responding most favorably, and what is the eco-physiological basis for their response to light? We hypothesized that as a forest understory growth of *C*. *nitidissima* would respond most favorably at low to intermediate light by optimizing photosynthetic activity, and high light will affect photosynthetic functions due to photoinhibition, damage of photosynthetic apparatus and concomitant enzyme activity.

**Results:**

With increasing light, the maximum net photosynthetic rate (*P*_Nmax_) and apparent quantum yield (AQY) decreased, while the light compensation point increased, and light saturation point first increased followed by a decrease. The *P*_Nmax_ and AQY under 50 and 100% sunlight were significantly lower than that under 10 and 30% sunlight. The chlorophyll fluorescence parameters *F*_m_, *F*_v_, *F*_v_/*F*_m_ all decreased under high light (> 50%). The contents of chlorophyll a (Chla), chlorophyll b (Chlb), and carotenoid (Car) decreased with increasing light. Relative conductivity, malondialdehyde (MDA) and proline contents in leaves were significantly increased in high light but we found no significant difference in these indices at 10 and 30% sunlight.

**Conclusions:**

We conclude that *C. nitidissima* is a shade adapted plant with poor adaptability to high light (> 50%). The novelty of this research is the demonstration of the eco-physiological basis of its light tolerance (conversely, shade adaptation) mechanisms indicated by decreased photosynthetic activity, chlorophyll fluorescence, Chla, Chlb and Car contents and concomitant increase in relative conductivity, MDA and proline contents at high light causing photoinhibition. For artificial propagation of *C. nitidissima* we recommend growing cuttings below 30% sunlight. For in situ conservation of this valuable, rare and endangered shrub it is necessary to protect its natural habitats.

## Background

*Camellia nitidissima* (Theaceae) is a rare and endangered evergreen shrub/small tree. It is one of the few *Camellia* species with yellow flowers. Because of its high aesthetic, cultural and germplasm value it is also called ‘‘the Queen of Camellia’’ [1, 2]. It has been introduced to Japan, Australia, Europe and North America as an ornamental plant and drew attention from horticulturists worldwide as a valuable genetic resource [3, 4]. It has a narrow distribution in Guangxi Province, South China and North Vietnam [5], growing as understory in evergreen broad-leaf forests along moist valley 50–650 m above sea level [6]. In China, *C. nitidissima* has been found only in two disjunctive areas in Guangxi Province, (i) at the junction of Fushu, Longan, and Fusui near Nanning city, and (ii) in Fangcheng, south of Mount Shiwan [7]. *C. nitidissima* is used as a Chinese traditional medicine [1]. Recently tea and other beverages made from its leaves and flowers have been commercialized and sold in China and Southeast Asia [8, 9]. Unfortunately, due to habitat loss and excessive collecting of seedlings in recent decades, its natural populations have declined dramatically. This species is now listed as one of the most endangered plant species and given protection in China [10].

Light is a predominant environmental factor affecting plant growth and development. Therefore, understanding plant response to light has been a long-term focus of plant eco-physiological research [11]. In optimal light conditions, both photosynthetic fixation of CO_2_ and rate of photosynthesis increase with increased absorption of light energy by chlorophyll, whereas excessive solar radiation suppress photosynthesis and may cause oxidative damage to the photosynthetic system [12–14]. Recent research on eco-physiology of rare and endangered plants emphasizes the study of the relationship between photosynthesis and incident light. Both deep shade and full light greatly reduce survival of English yew seedlings but 30% light seems to be optimal for its growth and development [15]; the maintenance of this species in European temperate forests depends mainly on selective canopy opening to reduce light competition [16]. Another endangered species *Lindera melissifolia* is a facultative shade plant. Although it has the ability to adapt to a range of light conditions it grows optimally below 40% light and hence this level of light has been suggested for its artificial propagation and reintroduction [17, 18]. The light stress brought by habitat fragmentation has been suggested as an important cause behind the extinction of seven-son flower (*Heptacodium miconioides*) and the species is adapted to moderate light intensity, 350–716 μmol m^−2^ s^−1^ [14]. Growth of *Arundinaria gigantea* is enhanced with increased light levels, and in this case reduction of overstory canopy has been suggested as a potential management tool for enhancing survival and growth of existing populations of this endangered species [19]. The limited ability of *Abies alba* saplings to exploit high-light conditions may be a competitive disadvantage in large canopy gaps limiting recruitment of this species to small gaps [20]. All these studies provide scientific basis for experimenting with rare and endangered species to determine their ecophysiological and growth response to light to device methods for ex situ propagation, conservation and recovery of endangered plants.

It has been found that light adaptability of *C. nitidissima* is narrow, and light environment is of critical importance to its survival, growth and development [21, 22]. In its natural habitats *C. nitidissima* grows as a forest understory with > 75% canopy cover [10]. As removal of upper canopy trees expose them to full sunshine, leaves of *C. nitidissima* turn yellow, branches gradually wither, and the plants eventually die [23]. Wei et al. [24] compared the differences of photosynthetic characteristics in *C. nitidissima* and its widespread congener *C. sinensis.* They concluded that *C. nitidissima* is a shade tolerant species, while *C. sinensis* has a wide range of adaptability to light making it more widely distributed. Qi et al. [25] measured photosynthesis light response of *C. nitidissima* in different seasons using 17 year-old plants grown in ex situ conservation habitats. They found that the species has the higher photosynthetic ability in autumn. Because younger seedlings and stem cuttings of *C. nitidissima* are used for artificial propagation it is necessary to test their growth response to varying light conditions. It is also necessary to measure additional physiological parameters to determine what level of light is appropriate for optimal growth and photosynthetic activity of this plant. Despite its rarity, endangered status, conservation value, and high demand for its medicinal and horticultural value the eco-physiological response of this plant to varying light levels has not been studied. It is necessary to understand its adaptability to a range of light conditions and how that is related to its growth. This knowledge will help devise effective methods for horticultural propagation using appropriate shade treatment above the stem cuttings. It will also help identify natural habitats for planting artificially propagated cuttings for restoration. Hence the main objective of this study was to investigate the responses of the photosynthesis and other physiological characteristics of *C*. *nitidissima* cuttings to a simulated light gradient created by artificial shading and analyze its sensitivity to light in order to provide a theoretical and practical basis for its ex situ conservation, artificial propagation and population recovery. Our specific research question was: At what light level will *C*. *nitidissima* cuttings respond most favorably, and what is the eco-physiological basis for their response to light? We hypothesized that as a forest understory shrub cuttings of *C*. *nitidissima* would respond most favorably at low to intermediate levels of light by optimizing photosynthetic activity compared to high light; high light (> 50%) will affect photosynthetic functions due to photoinhibition, damage to photosynthetic apparatus and concomitant enzyme activity.

## Methods

### Study site

The experiment was conducted at the Guangxi Institute of Botany (25°11′N, 110°12′E; 178 m a.s.l.), Guilin, Guangxi, South China. This region enjoys subtropical monsoon climate. The mean annual temperature is 19.2 °C, average temperature of the hottest and coldest months are 28.4 and 7.7 °C, respectively with extremes of 40 and − 6 °C, the number of months with average temperature above 20 °C is 6–7, the annual accumulated temperature above 10 °C is 5955.3 °C. Annual rainfall is 1854.8 mm, 73% of which falls between April and August. The relative humidity is 78.0% with distinct wet and dry season. Average annual sunshine is about 1550 h, and days with frost ranges from 9 to 24.

### Experimental material and treatment

**Fig. 1:**
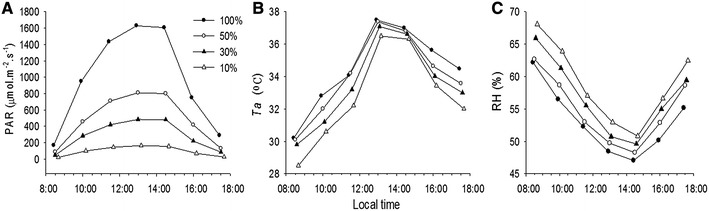
Diurnal variation of environmental factors under 10, 30, 50, and 100% sunlight, respectively. **A** Photosynthetically active radiation (PAR), **B** air temperature (*T*_a_), **C** relative humidity (RH)

We simulated a gradient of low to high light by filtering natural sunlight using black nylon that allowed 10, 30, 50 and 100% of full sunlight. Individual 2-years-old stem cuttings of *C*. *nitidissima* were used in this study. The cuttings were planted in plastic pots (30 cm inner diameter and 25 cm deep) containing 6 kg of soil (in a mixture of yellow clay, grass ash and pig manure in a ratio of 4:2:1). At the start of the shade treatment the cuttings were 18 to 22 cm tall each with 6–8 leaves. Eighty uniform potted cuttings were placed in a shade canopy that allowed 10% sunlight for a month of recovery/adjustment before the start of the experiment. The cuttings were divided randomly into four groups (20 pots/group) and moved to the pre-set shed canopies allowing 10, 30, 50 and 100% sunlight in early May, 2015. The pots were watered adequately at each dusk and compound fertilizers applied once a month. Photosynthesis and other physiological parameters were measured 2 months after the commencement of the shade treatments when the cuttings were well adjusted to the experimental shade gradient. Additionally, we measured the diurnal variation of photosynthetically active radiation (PAR), air temperature (*T*_a_) and relative humidity (RH) under each shade canopy at a clear day during the experiment (Fig. 1).

### Measurements

#### Light response curves

Light response curves were determined using the LI-6400 portable photosynthesis system (*LI*-*COR*, Lincoln, Nebraska, USA). Measurements were made on the upper, mature and fully expanded leaves from four randomly selected individuals in each treatment, each individual measured only once. To fully activate the photosynthetic system, the leaves were put under 400 μmol m^−2^ s^−1^ with the red and blue radiation source in the photosynthesis system for 20 min, then photosynthesis with an open air source was measured at flow rate of 500 cm^3^ min^−1^, leaf temperature 28 °C, CO_2_ concentration 360 μmol mol^−1^ (controlled by CO_2_ cylinder), and irradiances 1800, 1500, 1200, 1000, 800, 600, 400, 300, 200, 150, 100, 50, 20, and 0 μmol m^−2^ s^−1^ [26]. Each measurement was made for 2 min at each irradiance level on the upper fully expanded leaves of *C*. *nitidissima* cuttings in sunny days during 8:30–11:30 am. Maximum photosynthetic rate (*P*_Nmax_), apparent quantum yield (AQY), light saturation point (LSP), and light compensation point (LCP) were obtained by light response curve.

#### Chlorophyll fluorescence

Chlorophyll fluorescence was determined using a LI-6400 portable photosynthesis system fitted with a 6400–40 leaf chamber fluorometer. Eight to ten leaves per treatment were selected for the measurements. Minimal fluorescence (*F*_0_) of leaves fully exposed to darkness (more than 30 min) was determined under low light, after which the leaf was exposed to a saturate pulsed light (6000 μmol m^−2^ s^−1^, duration 0.8 s) to determine the maximum fluorescence (*F*_m_); then the variable fluorescence *F*_v_ = *F*_m_ − *F*_0_ and maximal quantum yield of PSII photochemistry (*F*_v_/*F*_m_) were calculated. All measurements were taken in the morning (7:00–9:00).

#### Photosynthetic pigments

After the photosynthetic measurements, four cuttings per treatment were selected to determine the photosynthetic pigments. Leaf chlorophyll (Chl) and carotenoid (Car) contents were determined following the method of Lichtenthaler [27]. The pigments were extracted with 95% ethanol, and the absorbance of extracted liquids was recorded at 665 and 649 nm for Chl, and at 470 nm for Car in a spectrophotometer (TU1901, Beijing Purkinje General Instrument Co., Ltd., China), based on which the contents of Chla, Chlb, and Car were calculated with formulas: Chla = 13.95 *A*_665_ − 6.88 *A*_649_, Chlb = 24.96 *A*_649_ − 7.32 *A*_665_, Car = (1000 *A*_470_ − 2.05 Chla − 114.8 Chlb)/245, as well as the ratios of Chla/b and Car/Chl.

#### Relative conductivity, malondialdehyde and proline contents

Because high relative conductivity, malondialdehyde (MDA) and proline content indicate damage to cell membrane and oxidative stress we measured these parameters in response to the light gradient [14, 28]. Fresh 1.0 g leaf samples were minced into small fragments, and incubated under vacuum condition for 15 min, then soaked in a glass tube with 20 mL distilled water. After 4 h extraction, the conductivity values were measured using a digital conductivity meter DDS-11A (Shanghai Optical Instrument Factory, China). After that, the samples were boiled for 20 min and conductivity was measured again after cooling the samples to room temperature. The relative conductivity was expressed as the ratio of the former conductivity to the corresponding latter [29]. Lipid peroxidation was determined by measuring MDA content according to the method of Hodges et al. [30]. First, 0.2 g fresh leaf tissue was homogenized in 10 mL 10% trichloroacetic acid (w/v). The homogenate was centrifuged at 4000×*g* for 10 min, and then 2 mL of the supernatant were mixed with 2 mL 0.67% 2-thiobarbituric acid (w/v). The mixture was incubated in boiling water (95–100 °C) for 30 min and then centrifuged at 4000×*g* for 10 min. The absorbance of reaction supernatant was measured at 450, 532, and 600 nm, and level of lipid peroxides was calculated following the formula: *C* (μmol/L) = 6.45 (*A*_532_ − *A*600) − 0.56 *A*_450_. The amount of proline was measured based on the method of Bates et al. [31]. Briefly, 0.2 g fresh leaf was homogenized in 10 mL 3% aqueous sulfosalicylic acid and filtered through filter paper. Then, 2 mL of the filtrate was mixed with 2 mL acid-ninhydrin and 2 mL glacial acetic acid and heated at 100 °C for 30 min, then 4 mL toluene was added to the mixture and the contents of tubes were stirred for 30 s. Absorbance of the red upper phase was measured at 520 nm. A standard curve for proline was constructed to determine the proline concentration in each sample.

#### Data analyses

Light response curves were fitted by modified rectangular hyperbola model [32–34].

$$ P_{N} (I) = \frac{\alpha (1 - \beta I)}{1 + \gamma I}I - R_{\text{d}} $$

where *P*_N_ is net photosynthesis, *I* is incident photosynthetic photon flux density, *R*_d_ is dark respiration, α is initial slope of the curve, β and γ are coefficients which are independent of *I*.

*P*_Nmax_ was calculated by $P_{N\text{max} } = \alpha \left( {\frac{{\sqrt {(\beta + \gamma )} - \sqrt \beta }}{\gamma }} \right)^{2} - R_{\text{d}}$.

LCP using the model ${\text{LCP}} = \frac{{ - (\gamma R_{\text{d}} - \alpha ) - \sqrt {(\gamma R_{\text{d}} - \alpha )^{2} - 4\alpha \beta R_{\text{d}} } }}{2\alpha \beta }$.

LSP using the model ${\text{LSP = }}\left[ {\sqrt {{{(\beta + \gamma )} \mathord{\left/ {\vphantom {{(\beta + \gamma )} \beta }} \right. \kern-0pt} \beta }} - 1} \right]/\gamma$.

The apparent quantum yield (AQY) was calculated as the slope of the linear regression of the light response curve below 100 μmol m^−2^ s^−1^.

All statistical analyses were performed using the software statistical package SPSS 18.0 (Chicago, IL, USA). We determined what level of light is appropriate for optimal growth and photosynthetic activity of this plant by comparing photosynthetic parameter, chlorophyll fluorescence, photosynthetic pigments, and other physiological parameters of the cuttings grown under the four levels of light. Differences between the treatments were determined by ANOVA followed by Duncan’s multiple-range test at the *P* < 0.05 level.

## Results

### Photosynthetic parameters

**Fig. 2:**
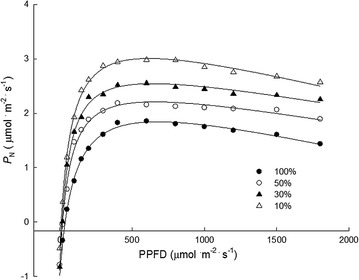
The photosynthetic light response curves of *C*. *nitidissima* grown under 10, 30, 50 and 100% sunlight, respectively

**Fig. 3:**
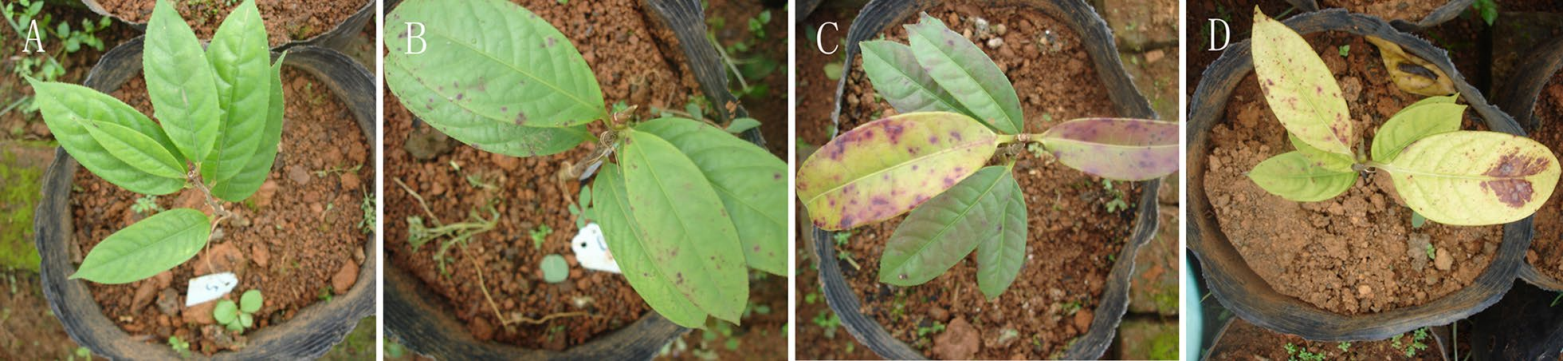
Seedling growth and leaf morphology of *C. nitidissima* grown under 10% (**A**), 30% (**B**), 50% (**C**) and 100% (**D**) sunlight, respectively

Under the same incident photosynthetic photon flux density, *C. nitidissima* grown under low light showed higher net photosynthesis rate (*P*_N_) than under high light (Fig. 2). The *P*_Nmax_ decreased with increasing light intensity (Table 1), *P*_Nmax_ under 30, 50 and 100% sunlight was decreased by 11.29, 21.92 and 38.41%, respectively, compared to that under 10% sunlight. LCP was increased with increasing light intensity while LSP first increased and then decreased. LCP was increased by 64.57, 99.44 and 278.97%, respectively, compared to that under 10% sunlight. LSP was highest at 50% sunlight. The AQY under 50 and 100% sunlight were significantly lower than that under 10 and 30% sunlight (Table 1). With increasing light intensity, *C. nitidissima* exhibited serious leaf discoloration. Under 30% sunlight, there were few brownish spots on leaves but the cuttings grew normally, under 50% sunlight, many brown spots appeared and under 100% sunlight, leaves were seriously burned causing defoliation (Fig. 3).

**Table 1:** Response of gas exchange parameters of *C. nitidissima* grown under 10, 30, 50 and 100% sunlight

RI (%)	*P*_Nmax_ (μmol m^−2^ s^−1^)	AQY (μmol μmol^−1^)	LSP (μmol m^−2^ s^−1^)	LCP (μmol m^−2^ s^−1^)
10	2.91 ± 0.28a	0.038 ± 0.0028a	537.25 ± 48.71c	9.89 ± 1.06c
30	2.58 ± 0.23ab	0.037 ± 0.0031a	706.57 ± 51.41a	16.28 ± 2.05b
50	2.27 ± 0.21b	0.027 ± 0.0025b	750.25 ± 52.83a	19.73 ± 2.65b
100	1.79 ± 0.17c	0.024 ± 0.0022b	619.50 ± 50.65b	37.50 ± 3.43a

Values are mean ± SD. Small letters indicate significant difference in mean value (± SD) of the parameters under different light levels (*P* < 0.05)

*RI* relative irradiance, *P*_Nmax_ maximum net photosynthetic rate, *AQY* apparent quantum yield, *LSP* light saturation point, *LCP* light compensation point

### Chlorophyll fluorescence

With increasing light intensity, *F*_m_, *F*_v_ and *F*_v_/*F*_m_ were all decreased significantly, while *F*_0_ first increased followed by a decrease. *F*_m_, *F*_v_ and *F*_v_/*F*_m_ under 50 and 100% sunlight were significantly lower than that under 10% sunlight, but no significant difference was observed between 10 and 30% sunlight. *F*_0_ at 100% sunlight was significantly lower than that under 10, 30 and 50% sunlight, with no significant difference among the latter three treatments (Table 2).

**Table 2:** Chlorophyll fluorescence characteristics of *C. nitidissima* grown under 10, 30, 50 and 100% sunlight

RI (%)	*F*_0_	*F*_m_	*F*_v_	*F*_v_/*F*_m_
10	182.07 ± 7.72a	940.67 ± 40.60a	758.60 ± 36.85a	0.806 ± 0.008a
30	193.37 ± 6.91a	885.89 ± 38.66a	692.51 ± 36.96a	0.782 ± 0.010ab
50	174.84 ± 31.47a	701.42 ± 68.17b	526.58 ± 49.43b	0.751 ± 0.032b
100	103.27 ± 21.95b	272.50 ± 55.42c	169.23 ± 34.74c	0.621 ± 0.023c

Small letters indicate significant difference in mean value (± SD) of the parameters under different light levels (*P* < 0.05)

### Contents of photosynthetic pigments and their ratios

**Fig. 4:**
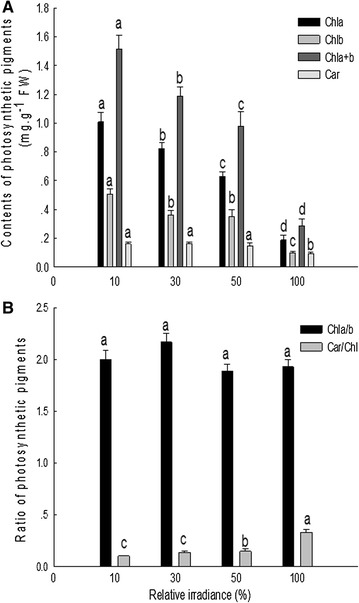
Contents (**A**) and ratio (**B**) of photosynthetic pigments in leaves of *C. nitidissima* grown under 10, 30, 50 and 100% sunlight, respectively. Different letters above the histograms indicate significant difference among different light levels (*P* < 0.05)

Leaf Chla, Chlb, Chla+b and Car contents of *C. nitidissima* decreased significantly with increasing light intensity. Chla contents in plants grown under 30, 50 and 100% sunlight were decreased by 18.53, 37.66 and 81.47%, Chlb contents decreased by 28.26, 31.03 and 80.83%, Chla+b contents decreased by 21.78, 35.45 and 81.25%, and Car contents decreased by 2.50, 10.00 and 41.87%, respectively, compared to that in cuttings grown under 10% sunlight (Fig. 4A). The ratio of Car/Chl showed an increasing trend with increasing light, while Chla/b showed no significant difference among the light treatments (Fig. 4B).

### Relative conductivity, MDA and proline contents

**Fig. 5:**
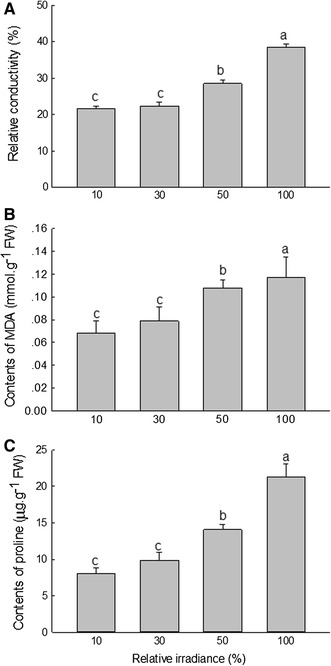
The relative conductivity (**A**), MDA (**B**), and proline (**C**) contents in leaves of *C*. *nitidissima* grown under 10, 30, 50, and 100% sunlight, respectively. Different letters above the histograms indicate significant difference among different light levels (*P* < 0.05)

With increasing light intensity there was a significant increase in relative conductivity and MDA contents of *C. nitidissima*. These two indices under 50 and 100% sunlight were significantly higher than that under 10% sunlight. However, there was no significant difference in these parameters between cuttings grown under 10 and 30% sunlight (Fig. 5A, B). Proline contents in plants grown under 30, 50 and 100% sunlight were 1.22, 1.74 and 2.63 times than those under 10% sunlight (Fig. 5C).

## Discussion

Light is a primary energy source necessary for photosynthesis, growth and development of plants. However, when light absorbed by leaves cannot be optimally exploited or dissipated, plants suffer from light stress. This may cause reduction in photosynthetic function, photoinhibition or even light oxidation, leading to destruction of photosynthetic apparatus [13, 35]. In this study, we found that *P*_Nmax_ of *C. nitidissima* was 2.91 μmol m^−2^ s^−1^, LSP 537.25 μmol m^−2^ s^−1^, and LCP 9.89 μmol m^−2^ s^−1^ under 10% sunlight, significantly lower than its widespread congener *C. sinensis* (*P*_Nmax_ = 10.16 μmol m^−2^ s^−1^, LSP = 768.00 μmol m^−2^ s^−1^, and LCP = 10.76 μmol m^−2^ s^−1^) [22] and *C. oleifera* (*P*_Nmax_ = 9.71 μmol m^−2^ s^−1^, LSP = 1427.00 μmol m^−2^ s^−1^, and LCP = 19.60 μmol m^−2^ s^−1^) [36], indicating that it is a shade adapted plant responding negatively to high light, which supports our hypothesis.

Leaf-level photosynthetic characteristics have been widely used as a tool for detecting environmental stress and for determining growth conditions suitable for different plants [37]. In this study we found that *P*_Nmax_ of *C. nitidissima* decreased by 11.29, 21.92 and 38.41%, respectively, under 30, 50 and 100% sunlight compared to 10% sunlight, indicating that photosynthesis was inhibited under high light. Because of decreased chlorophyll content under high light the leaves capture less light. High light may also cause damage to PSII structure, reduce rubisco enzyme activity, increase dark-respiration and photorespiration, which in turn may lead to decreased photosynthetic rate [38]. This result was similar to other shade tolerant plants showing reduced photosynthetic capacity in high light; but there are also some differences: The *P*_Nmax_ of *C. nitidissima* was highest in low light (10% sunlight) while other shade tolerant plants showed highest *P*_Nmax_ in medium light (30–50% sunlight) [39–41], indicating that *C. nitidissima* appears to be an obligatory shade species with limited acclimation ability to high light conditions [42–44]. AQY is an important parameter to reveal photochemical efficiency, plants grown without environmental stress, the AQY range is 0.03–0.05 μmol μmol^−1^ [45]. In our experiment, the AQY of the cuttings grown under 50 and 100% sunlight were both lower than 0.03, indicating that *C. nitidissima* grown under these two light levels were subjected to some degree of photoinhibition. In this study, we found an increase of 1.65×, 1.99×, and 3.79× under 30, 50, and 100% sunlight compared with 10% sunlight for LCP of *C. nitidissima*. The elevated LCP may be attributed to lower chlorophyll content per unit leaf area and higher dark and light respiration under high light, which resulted in weakened light-harvesting capability and increased carbon assimilation. This is consistent with many other plants whose LCPs were found to increase with increasing irradiance [15, 46]. In our study, with increasing light intensity, the LSP first increased followed by a decrease, which indicates that *C. nitidissima* has poor adaptability to high light, unlike sun plants having increased LSP with increasing light intensity [47, 48]. These and other results discussed below also support our hypothesis.

Measurement of chlorophyll fluorescence is a non-invasive, rapid, and quantitative method of assessing the properties of photosynthetic apparatus and the extent to which plants are affected by different types of environmental stress [49, 50]. In this experiment, with increasing light intensity, *F*_0_ values first increased followed by a decline. The *F*_0_ value increase under 30% sunlight may be related to inactivation of PSII reaction centers, while its decline under 50 and 100% sunlight may be predominantly attributed to decreased chlorophyll content. *F*_m_ and *F*_v_ of *C. nitidissima* decreased significantly under 50 and 100% sunlight compared to 10% sunlight, indicating the inactivation and/or damage to PSII center complex under high light [51]. The *F*_v_/*F*_m_ is widely used as an indicator of photoinhibition [52, 53]. Under normal physiological conditions, the *F*_v_/*F*_m_ values of the vast majority of C_3_ plants range between 0.8 and 0.84. When the *F*_v_/*F*_m_ value of a plant is below this range, the plant is exposed to environmental stress [54, 55]. In this experiment, the *F*_v_/*F*_m_ of *C. nitidissima* under 50 and 100% sunlight were 0.751 and 0.621, respectively, indicating that the cuttings grown under these two light levels suffered from long-term photoinhibition. The photosynthetic apparatus presumably absorbed excessive light, resulting in the inactivation or impairment of the PSII reaction centers [41]. This result is consistent with the shade plants *Eugenia uniflora* and *Lasianthus attenuates*, the *F*_v_/*F*_m_ of these plants decreased significantly without complete recovery for a long time when they are transferred from shade to high light [44, 53]. However, the *F*_v_/*F*_m_ value under 30% sunlight was close to 0.8, which suggests that the PSII reaction center functioned normally and the level of photoinhibition was not high.

Chlorophyll absorbs light energy in photosynthesis and chlorophyll content is directly related to rate of photosynthesis [56]. Changes in the Chla/b ratio are related to the balance of light absorption capacity of photosystems [57]. Car/Chl ratio reflects the relationship between light absorption and light damage protection in plants [58]. In this experiment, Chl content (Chla, Chlb, and Chla+b) of *C. nitidissima* decreased significantly with increasing light intensity. Under 100% sunlight, the contents of Chla, Chlb and Chla+b all decreased more than 80% than those in 10% sunlight, which was significantly higher than in *Tetrastigma hemsleyanum* and *Cypripedium guttatum* [41, 59], causing Chl bleaching. Such high light may seriously impair or totally inactivate the photosynthetic system. These results may partly explain the low photosynthetic rates of *C. nitidissima* grown under high light. Typically, plants grown in high light have higher Chla/b ratio than those in low light [59]. Increased Chl a/b ratios are in turn, associated with decrease in the size of the PSII light-harvesting antenna, and changes in Rubisco [60]. However, we found no significant difference in Chla/b under different light levels, meaning that *C. nitidissima* cannot adjust the relative contents of Chla and Chlb to acclimate in high light. It is possible that the increased Car/Chl ratio under high light decreased light absorption in leaves to protect the photosynthetic apparatus from light damage, a protection mechanism to cope with high light stress.

An increase in relative conductivity indicates damage to cell function and results from decomposition of cell membranes and infiltration of metal ions as consequence of stress [61]. The content of MDA, a product of lipid peroxidation, has been considered as an indicator of oxidative damage [62]. In this study, the relative conductivity and MDA contents of leaves under 50 and 100% sunlight were both significantly higher than those grown under 10% sunlight, which indicate that high light caused damage to the membrane system of *C. nitidissima* leaves, and photo-oxidation might have occurred. This response is similar to *H. miconioides* and *Monimopetalum chinense* which have much elevated relative conductivity and MDA content of leaves under high light [14, 40]. Under 30% sunlight, these two values were not significantly higher than that under 10% sunlight, which indicate that there was no oxidative damage at this level of light. These results are further substantiated by the high proline contents at 50 and 100% sunlight. The lack of acclimation capacity of *C. nitidissima* to increasing light is further supported by the early senescence of its leaves. As light intensity increased, leaf color changed from dark green to light green to yellow green, and leaf burning was clearly visible. Under 50% sunlight, many brown spots appeared and, under 100% sunlight, leaves were seriously burned and abscised.

In its natural habitats *C. nitidissima* grows in valleys and streamsides in shady and moist evergreen broad-leaf forests. Due to anthropogenic disturbance and environmental deterioration, its habitat has been destroyed and continuously fragmented. An altered microclimatic environment could directly influence plant growth and recruitment [63, 64]. One important consequence of habitat fragmentation is increased exposure of understory plants to light. Our results demonstrate that *C. nitidissima* has limited acclimation potential to high light, and increasing irradiance has a negative effect on its physiological functions, growth and survival. The changes in microclimate under fragmented habitats also include higher air and soil temperature, higher evaporation and desiccation, lower relative humidity and soil moisture than forest interiors, all causing drought stress [65, 66]. As *C. nitidissima* is a drought intolerant plant [67], these high light-mediated environmental stresses are contributing to further endangerment of this species. In natural conditions *C. nitidissima* grows under evergreen broad-leaf forests with light intensity less than 3% full sunlight [68]. Although *C. nitidissima* can grow normally below 30% sunlight in experimental condition, we do not recommend reducing the overstory canopy for in situ conservation of this species because that will increase competition from other plants that grow optimally under medium light intensity. For artificial propagation we recommend using shade structures that will allow 10–20% sunlight above the cuttings.

## Conclusions

Based on our results, we conclude that *C. nitidissima* is a shade adapted plant with poor adaptability to high light environment. The plant showed no obvious photoinhibition under 30% sunlight, while under 50 and 100% sunlight, respectively, it experienced serious photoinhibition and the photosynthetic apparatus was damaged likely due to photo-oxidation. This is indicated by decreased *P*_Nmax_, AQY, *F*_m_, *F*_v_, *F*_v_/*F*_m_, Chla, Chlb, Car contents and concomitant increase in relative conductivity, MDA and proline contents of leaves at high light (50 to 100%). The novelty of this research is that we demonstrate the ecophysiological basis of light tolerance (conversely, shade adaptation) mechanisms in *C. nitidissima*. For artificial propagation of cuttings of this plant we recommend growing cuttings below 30% sunlight. For in situ conservation of this valuable, rare and endangered shrub it is necessary protect its natural habitats.

## Abbreviations

PPFD: photosynthetic photon flux density
*P*_N_: net photosynthetic rate
PAR: photosynthetically active radiation
*T*_a_: air temperature
RH: relative humidity
LCP: light compensation point
LSP: light saturation point
AQY: apparent quantum yield
*P*_Nmax_: photosynthetic rate at irradiation saturation
*F*_0_: minimal fluorescence
*F*_v_: variable fluorescence
*F*_m_: maximum fluorescence
*F*_v_/*F*_m_: maximum quantum yield of PSII
Chl: chlorophyll
Car: carotenoids
MDA: malondialdehyde
